# A time-based double-sided readout concept of 100 mm LYSO:Ce,Ca fibres for future axial TOF-PET

**DOI:** 10.1186/s40658-023-00563-6

**Published:** 2023-07-14

**Authors:** Konstantin Weindel, Vanessa Nadig, Katrin Herweg, Volkmar Schulz, Stefan Gundacker

**Affiliations:** 1 Department of Physics of Molecular Imaging Systems, Institute for Experimental Molecular Imaging, RWTH Aachen University, Aachen, Germany; 2 Physics Institute III B, RWTH Aachen University, Aachen, Germany; 3 Hyperion Hybrid Imaging Systems GmbH, Aachen, Germany

**Keywords:** PET, Axial TOF-PET, DOI, Double-sided high-frequency readout

## Abstract

**Background:**

Positron emission tomography (PET) requires a high signal-to-noise ratio (SNR) to improve image quality, with time-of-flight (TOF) being an effective way to boost the SNR. However, the scanner sensitivity and resolution must be maintained. The use of axially aligned 100-mm LYSO:Ce,Ca scintillation crystals with double-sided readout has the potential of ground-breaking TOF and sensitivity, while reducing parallax errors through depth-of-interaction (DOI) estimation, and also allowing a reduction in the number of readout channels required, resulting in cost benefits. Due to orientation, these fibres may also facilitate the integration of TOF-PET with magnetic resonance imaging (MRI) in hybrid imaging systems. The challenge of achieving a good spatial resolution with such long axial fibres is directly related to the achievable TOF resolution. In this study, the timing performance and DOI resolution of emerging high-performance materials were investigated to assess the merits of this approach in organ-dedicated or total-body/large-scale PET imaging systems.

**Methods:**

LYSO:Ce,Ca scintillation fibres of 20 mm and 100 mm length were tested in various operating and readout configurations to determine the best achievable coincidence time resolution (CTR) and DOI resolution. The tests were performed using state-of-the-art high-frequency (HF) readout and commercially available silicon photomultipliers (SiPMs) from Broadcom Inc.

**Results:**

For the 100-mm fibre, an average CTR performance of $137 \pm 1$ ps FWHM and an average depth-of-interaction resolution within the fibre of $12.3 \pm 0.5$ mm FWHM could be obtained. The 20-mm fibre showed a sub-100 ps CTR of $98 \pm 1$ ps FWHM and a fibre resolution of $8.5 \pm 0.2$ mm FWHM in the double-sided readout configuration.

**Conclusion:**

With modern SiPMs and crystals, a double-sided readout of long fibres can achieve excellent timing resolution and field-advancing TOF resolution, outperforming commercial PET systems. With 100-mm fibres, an electronic channel reduction of about a factor 2.5 is inherent, with larger reduction factors conceivable, which can lead to lower production costs. The spatial resolution was shown to be limited in the axial direction with 12 mm, but is defined to 3 mm in all other directions. Recent SiPM and scintillator developments are expected to improve on the time and spatial resolution to be investigated in future prototypes.

## Background

Positron emission tomography (PET) has become one of the most important functional imaging techniques in the study of living organisms. An image is produced with the aid of $\beta ^+$ radioactive tracers. Positron-electron annihilation as a consequence of the tracer decay leads to the back-to-back emission of two 511-keV gamma photons on a so-called line of response (LOR). The identification of many of such LORs through gamma-photon detection enables the spatial localization of the tracer through image reconstruction [1].

Among others, PET can be used for organ-specific examination, for example, in the field of neurological research and diagnosis. Here, PET is used to study Alzheimer’s, Parkinson’s and other neurodegenerative diseases and has become a key modality for early-stage detection, treatment and fundamental studies on disease origin and onset in an ageing society. Furthermore, a major focus is put on the combination of PET systems with structural imaging techniques, such as magnetic resonance imaging (MRI), to provide high soft tissue contrast in so-called hybrid systems [2, 3]. In addition, there are attempts on the commercialization of total-body scanners, which can capture the entire human body in a single acquisition exploiting their large geometry to vastly increase sensitivity. Such high sensitivity of large-scale scanners allows the acquisition time to be reduced to just a few minutes, and additionally the image quality to be improved [2, 4]. Such systems might completely change the verdict of PET, due to the extremely low doses required and high sensitivities obtained, e.g. even allowing to visualize the working immune system [5, 6]. However, all of these approaches are in need of low-cost and straightforward detector designs, which is currently the pitfall when it comes to commercialization [4].

Current state-of-the-art systems use the time-of-flight (TOF) information of the two $\gamma$-photons of the same emission to further increase the accuracy and signal-to-noise ratio (SNR) [7, 8]. This additional information makes it possible to spatially constrain the event probability on every single LOR. The coincidence time resolution (CTR) describes the accuracy in time with which the difference in time of arrival of the photons can be resolved and is therefore the key parameter to indicate how accurately the position of annihilation along the LOR can be determined, or what gain $G$ of the SNR is achieved (compared to a non-TOF system) [9]:

1 $$\begin{aligned} G = \frac{\text {SNR}_\text {TOF}}{\text {SNR}_\text {non-TOF}} \sim \sqrt{\frac{2D}{c_0 \ \text {CTR}}}. \end{aligned}$$

In Eq. (1), $D$ is the diameter of the imaged object, $c_0$ the speed of light in vacuum and the $\text {CTR}$ is the temporal resolution of the scanner. The gain is therefore greatest for larger-sized objects, as there is more background noise for TOF to reject, and is improved by higher temporal resolutions for similar reasons [9, 10].

**Fig. 1:**
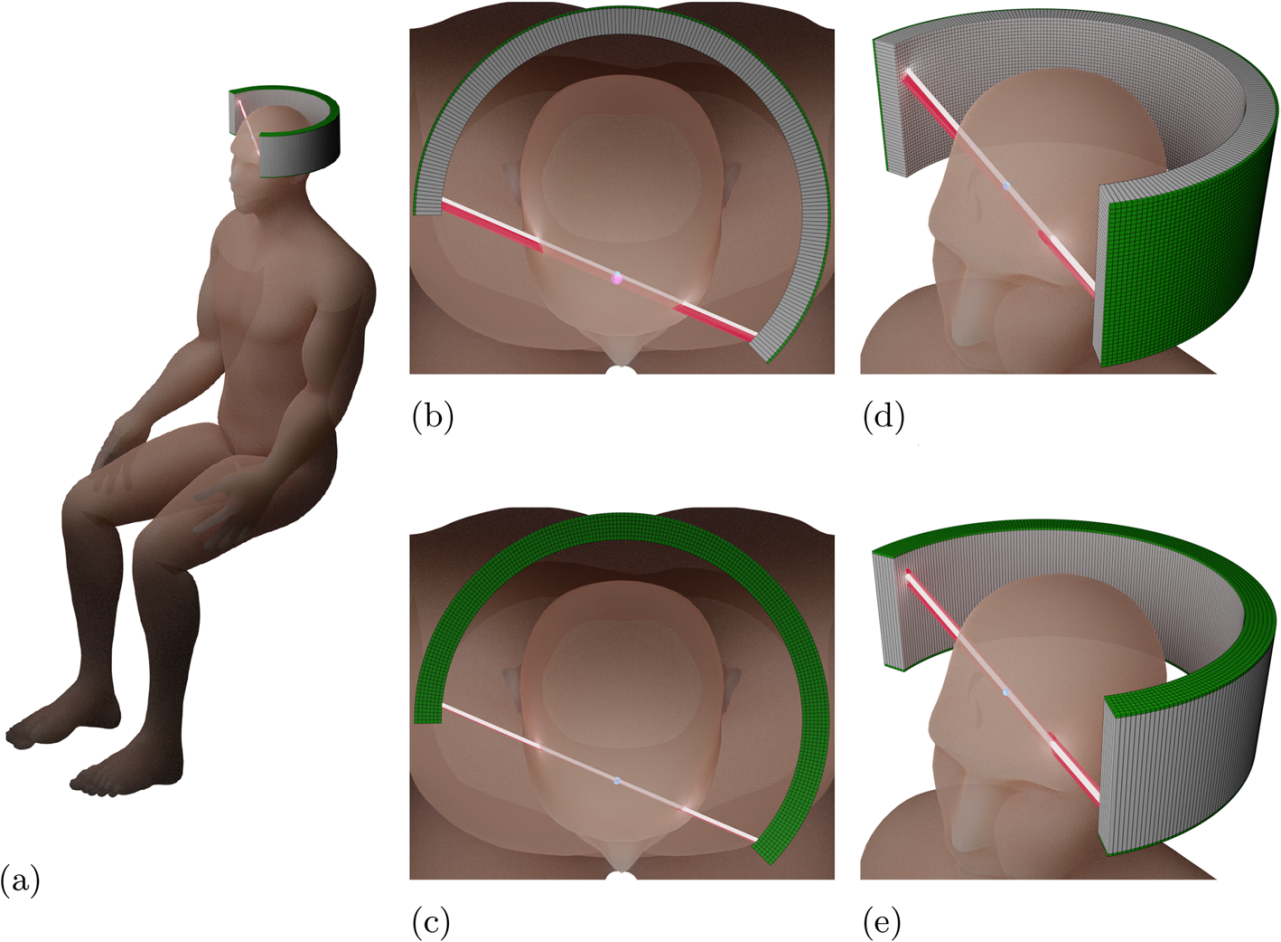
Brain PET prototypes in comparison: **b** and **d** show a brain PET constructed from conventional sizes of scintillators in single-sided readout (SiSi). In **a**, **c** and **e** are different views of the double-sided readout (DoSi) model. The detector ring is not shown in its entirety for illustrative purposes. The scintillation crystals are shown in grey, the SiPMs in green. The white line is the best possible resolution of the LOR and in red the error on the LOR determination due to parallax error. The human model used is based on [14]. **a** The detector ring is positioned around the head of a seated patient, **b** top view of a classical readout detector type, **c** top view of the novel readout detector type, **d** side view of a classical readout detector type, **e** side view of the novel readout detector type

Improving the sensitivity to true coincidences will significantly increase the SNR, allowing lower dosages and/or shorter scan times. However, increasing the thickness of scintillation crystals or gantry size can be costly, especially for total-body PET systems, and may also result in stronger parallax errors for the outer rings. This type of spatial distortion occurs when the annihilation photons produced by positron-electron annihilation events are registered at different positions in the detector pixels. Geometrically, the effect is intensified towards the outer edges of classical PET systems. Alternatively, moving closer to the organ being imaged can also lead to parallax errors [1, 11], particularly in small systems such as brain PET (see Fig. 1a). Therefore, the exact 3D estimation of the gamma impact point in the scintillator is of utmost importance [12].

Even though organ-specific scanners and total-body PET systems work on completely different scales, they are united by the fact that both suffer from parallax errors and benefit from a reduction of readout channels in order to reduce costs. Current TOF-PET systems typically use $3 \times 3 \times 20 \text { mm}^{3}$ long scintillation crystals aligned radially to the gantry, each read out on their base area with silicon photomultipliers (SiPMs) (comparable to Fig. 1b, d) [1, 12]. In such systems, the temporal as well as the spatial resolutions deteriorate as the length of the crystals increases [13]. The first is due to the fact that the travel times of optical photons in the crystal become longer, and the second is due to the increasing influence of the parallax error. By encoding where the absorption took place within the scintillator, i.e. the determination of the depth of interaction (DOI), the latter effect can be compensated for and a higher spatial resolution can be achieved, even in the peripheral regions of the field-of-view (FOV). Especially in brain-PET, parallax errors are provoking large loss in resolution, due to the large solid angle coverage [1, 11].

There are several approaches to obtaining DOI information, of which double-sided readout (DoSi) is the most straightforward. For DoSi, both short ends of the scintillator are connected to a photodetector (SiPM), which takes advantage of the scintillation light absorption along the crystal, allowing to determine the DOI by the ratio of detected light intensities on both crystal ends. Such an approach, however, always compromises between achievable DOI and timing resolution (CTR) and furthermore does not allow for very long scintillating fibres. Another possibility to determine the location of the gamma absorption within the crystal is via the time-of-flight differences between both ends. Only recently, this approach has become possible as a consequence of the rapid development of analogue SiPM technology and crystal materials [15–18].

In this article, the potential of a detector concept that, on the one hand, makes use of this DoSi readout and, on the other hand, allows reducing the number of SiPMs and readout channels used is investigated. A sole time-based gamma interaction estimation method will be studied, similar to concepts discussed in [19–22]. For this purpose, the scintillation crystals are extended to 100 mm in length and then aligned axially to the gantry (as depicted in Fig. 1a, c, e). A similar approach was superficially explored in [19], using digital SiPMs on LYSO crystals, but not tested with newly developed analogue SiPMs, high-frequency (HF) readout and LYSO:Ce,Ca crystals, which have been shown in literature to break current timing records [16–18, 23, 24].

With such a detector architecture, a reduction of the used SiPMs by a factor of 2.5 compared to conventional systems would be possible. This detector concept would further allow for easy implementation of various multiplexing schemes enabling additional channel compression. Another advantage of the axial alignment is that it opens up interference-free windows for MRI magnetic fields in-between the PET electronics. The detector tested in this study uses HF electronics for the SiPM signal readout [17, 24, 25]. To find out the resolution limits of such a detector concept, several measurements have been conducted, using solely commercially available state-of-the-art detector material.

## Methods

**Fig. 2:**
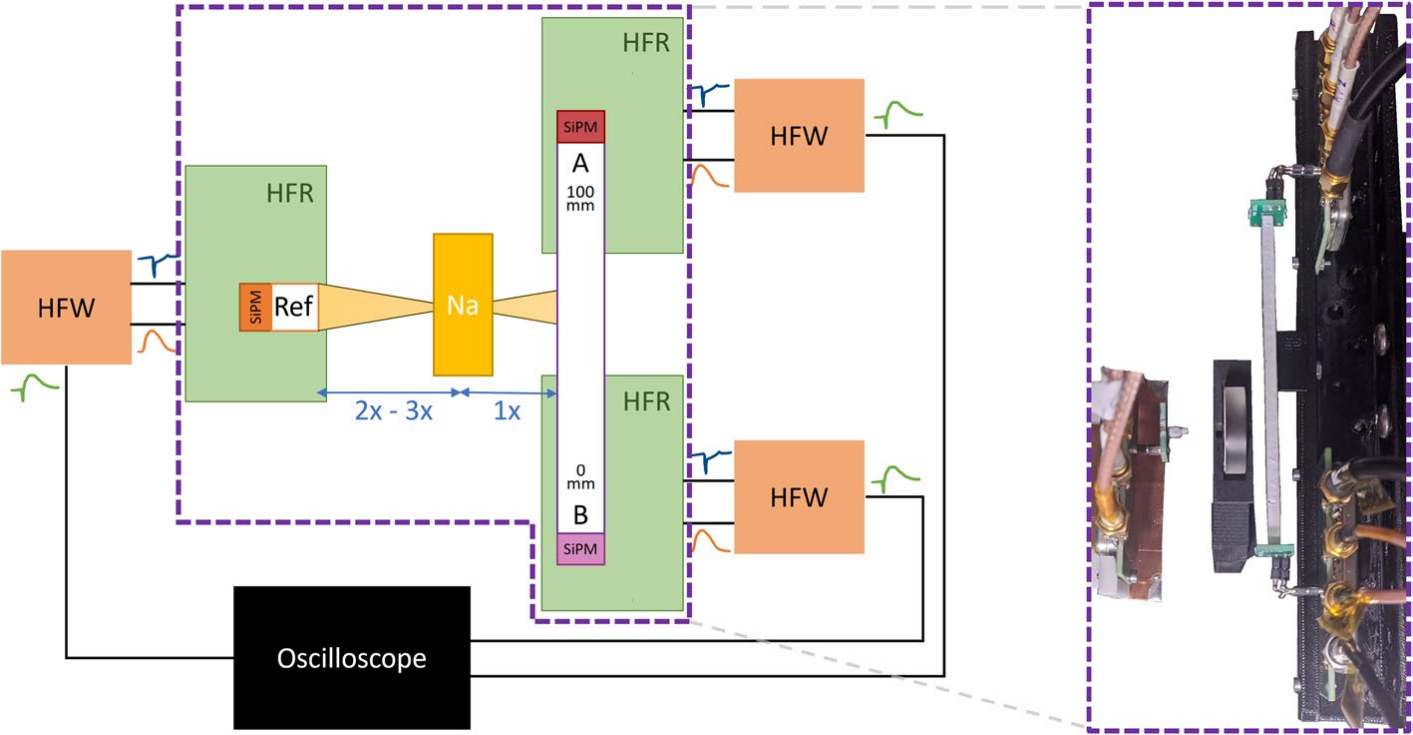
Sketch of the experimental setup with the 100-mm fibre and picture of that same setup, test source (${}^{22}$Na) and high-frequency readout boards (HFRs) inside a temperature controlled test chamber (dashed frame)

The coincidence time resolution test setup can be seen in Fig. 2. Each detection unit consists of a scintillation detector, a scintillation crystal in combination with an SiPM, and an HF readout board that generates the timestamp upon event detection and measures the corresponding energy deposit [17, 25]. The data processing, such as coincidence time difference determination and energy integration of the signals, is done by means of an oscilloscope (LeCroy waverunner 9404M-MS).

### Detector prototype using state-of-the-art scintillators and SiPM technology

Commercially available inorganic lutetium-yttrium oxyorthosilicate crystals double-doped with cerium and calcium (LYSO:Ce,Ca) manufactured by Taiwan Applied Crystals [26] have been evaluated in our laboratory and were chosen due to their high light yield and fast decay time along with excellent optical properties [18, 27].

All measurements were performed against a $2 \times 2 \times 3 \text { mm}^{3}$ LYSO:Ce,Ca reference crystal with four faces polished. DOI measurements were carried out on two different scintillator lengths, namely a $3 \times 3 \times 20 \text { mm}^{3}$ and a $3 \times 3 \times 100 \text { mm}^{3}$ fibre, both with all six crystal faces polished. The crystals were cleaned with isopropanol before use and all lateral sides covered with Teflon tape (at least 4 layers) to minimize possible light losses.

The scintillation photons were detected by Broadcom AFBR-S4N33C013 SiPMs [28] optically coupled to the crystals. The SiPMs feature an active area of $3\times 3 \text { mm}^{2}$, which exactly matches the base area of the scintillation fibres, and a single photon avalanche diode (SPAD) size of $30 \times 30\, \mu \text {m}^{2}$. Cargille Meltmount^™^ [29] with a refractive index of $n_D({25\,}^{\circ }\hbox {C}) = {1.582}$ [30] was used as an optical coupling agent.

### Prototype readout using ultra-fast HF electronics

**Fig. 3:**
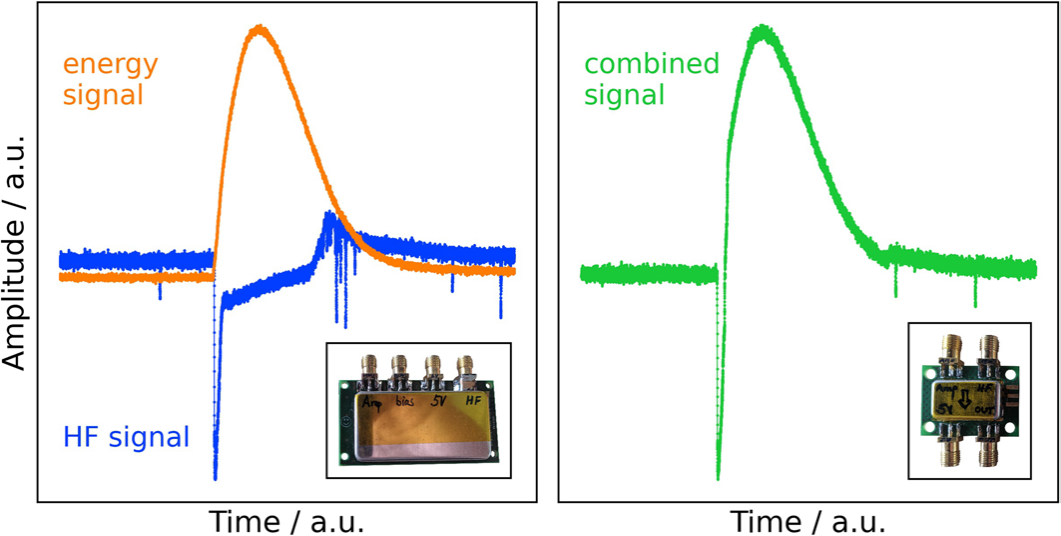
The HFwitch (HFW) board combines the fast timing signal (negative polarity) and an amplified energy signal (positive polarity) into one single signal

The SiPMs were electronically connected to HF readout boards, which have already been presented in the literature [17, 25]. The boards come with a timing and an energy output. The contribution of the HF electronics to the overall TOF resolution is negligibly small, such that it is possible to study the physical limits of the scintillator and SiPM materials. To operate all channels of the prototype detector with our Teledyne LeCroy waverunner 9404M-MS oscilloscope, which is limited to four channels, a combiner board (HFW) was developed that combines the two information-carrying pulses as shown in Fig. 3. The performance has been put to the test in several measurements and allows the use of the electronics without losses in timing resolution.

Measurements are carried out in a temperature-stabilized and light-protected test chamber at 16 ${^\circ }$C [31]. The scintillation detectors consisting of crystal and SiPM as well as their respective HF readout board and a ${}^{22}\hbox {Na}$ test source are located inside this chamber. Either a $3 \times 3 \times 100 \text { mm}^{3}$ (shown in Fig. 2) or $3 \times 3 \times 20 \text { mm}^{3}$ fibre is clamped on the right side or a second reference crystal is installed. DOI steps of 2 mm and 4 mm are made for the 20-mm and 100-mm fibre, respectively. The DOI resolution imposed by the setup has been computed from the setup geometry and is limited from 1.3 mm to 1.7 mm. It is not deconvolved from the measurement. As the DOI resolution imposed by the setup is significantly smaller than the DOI resolution obtained by the fibre time resolution  and the charge ratio, it is assumed that the DOI resolution of the setup does not impact the results reported.

In addition to the DOI scans, a series of measurements with single-sided (SiSi) readout of the 20-mm fibre was made for comparison. For all setups, scans at various threshold ($U_\text {thld}$) and bias operating voltages ($U_\text {bias}$) were conducted to show the optimal operation point of the respective detectors.

### Data analysis

As already discussed, the main objective is to determine the interaction position of the $\gamma$-photon along the scintillating fibre, using the time differences of the signals detected by the two SiPMs connected in double-sided readout. Two parameters are the figures of merit, i.e. the achievable time resolution of the fibre in a coincidence setup of the TOF-PET system, given by the coincidence time resolution (CTR), and the fibre time resolution (FTR), which describes the Gaussian delay time histogram given by the two SiPMs connected to the far ends of the fibre. The FTR is directly related to the achievable DOI resolution within the fibre.

The recorded delay time data were selected to photopeak events in a $\pm 2\sigma$ environment. The full width at half maximum (FWHM) of the pre-filtered TOF distribution served as the characterizing parameter of the time resolution, namely the CTR or FTR, depending on the SiPMs considered. Due to the measurement being conducted against a reference crystal, a correction against this reference CTR ($\text {CTR}_\text {ref}$) had to be done to determine the true CTR of a system consisting of only fibres [32]. The CTR measured ($\text {CTR}_\text {measured}$) in the experiment results from the FWHM of the Gaussian fit of the $\text {TOF}_{\text {measured}}$ distribution and is essentially the squared sum of the CTR of the reference and the CTR of the fibre ($\text {CTR}_\text {fibre}$):

2 $$\begin{aligned} \text {CTR}_\text {measured} = \sqrt{\frac{\text {CTR}_\text {fibre}^2}{2} + \frac{\text {CTR}_\text {ref}^2}{2}}. \end{aligned}$$

The $\text {CTR}_\text {fibre}$ is the CTR relevant for system comparability as it is independent of the influence of the reference CTR. Therefore, only the $\text {CTR}_\text {fibre}$ is used in the following and the subscript fibre is omitted:

3 $$\begin{aligned} \text {CTR}_\text {fibre} = \sqrt{2\cdot \left( \text {CTR}_\text {measured}\right) ^2 - \left( \text {CTR}_\text {ref}\right) ^2} \equiv \text {CTR}. \end{aligned}$$

For classical systems with only one pair of detectors, the desired CTR results directly. In the DOI experiment setup with three detectors used, a CTR between each of the two fibre ends (A and B) and the reference can be determined, as well as a CTR between both fibre ends, which is the FTR introduced above.

To determine the CTR of the whole fibre against the reference, the timing information of both fibre ends (A and B) was combined and averaged event-wise to obtain the required TOF distribution, as described in Eq. (4):

4 $$\begin{aligned} \Delta {t} = \frac{\Delta {t}_\text {A} \cdot E_\text {A} +\Delta {t}_\text {B} \cdot E_\text {B}}{E_\text {A}+E_\text {B}}. \end{aligned}$$

Here, $\Delta {t}_\text {A}$ and $\Delta {t}_\text {B}$ denote the time difference information between the respective SiPM coupled to the fibre and the reference SiPM ($\Delta t_X = t_X -t_\text {ref}$) and $E_\text {A}$ and $E_\text {B}$ are the energy information measured at the respective SiPM (compare Fig. 2). The energy weighting is taking into account the Fisher information of the timestamps, but in further considerations these values are also set to 0.5; hence, a calculation of the arithmetic mean (with equal weights) is applied.

The DOI determination via timing exploits the finite signal propagation speed within the scintillator. By comparing the timestamps of both SiPMs at the ends of the fibre ($\Delta t_\text {F} = t_A - t_B$), a conclusion can then be drawn about the absorption position for each $\gamma$-photon within the fibre. The FTR defines the resolution of this runtime information and is estimated again by the FWHM of the Gaussian fit to the histogram of the distribution. The axial DOI resolution ($\text {DOI}_\text {res}$) described in Eq. (5) can then be determined, where $\mu _\text {F}$ is the centroid of the Gaussian fit to the TOF distribution, which shifts with the DOI:

5 $$\begin{aligned} \text {DOI}_\text {res}^\text {T} = \left( \frac{\textrm{d}{\mu _\text {F}}}{\text {dDOI}}\right) ^{-1} \cdot \text {FTR}. \end{aligned}$$

Similarly, the DOI resolution can be determined from the energy signals, due to light absorption in the fibre. Instead of the FTR, the average FWHM of the two photopeaks in the energy histogram is used, as is the distance between the two photopeaks $\left( \mu ^\text {E}_\text {A}-\mu ^\text {E}_\text {B}\right)$:

6 $$\begin{aligned} \text {DOI}_\text {res}^\text {E} = \left( \frac{\textrm{d}{\left( \mu ^\text {E}_\text {A}-\mu ^\text {E}_\text {B}\right) }}{\text {dDOI}}\right) ^{-1} \cdot \langle \text {FWHM}\rangle ^\text {E}. \end{aligned}$$

## Results

### Single-sided readout of the 20-mm fibre

**Fig. 4:**
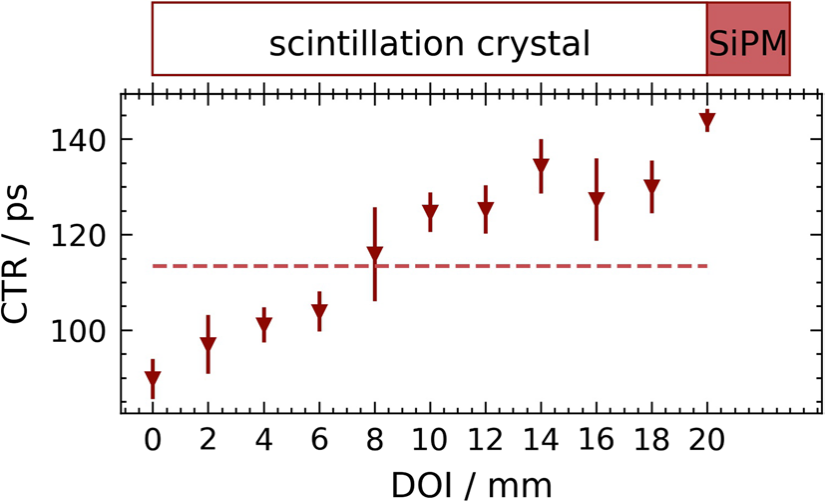
DOI-dependent CTR and the conventionally determined head-on CTR (shown as dashed line) in a 20-mm LYSO:Ce,Ca fibre. The position furthest from the SiPM, i.e. the end of the crystal that is not attached to the SiPM, is defined as position 0 mm and increases towards the SiPM until the complete length of 20 mm is reached at the point of contact with the SiPM

From classical head-on irradiations of the 20-mm LYSO:Ce,Ca scintillator at 32 V and 37 V bias voltage with varying threshold voltages, the best CTR was obtained for $U_{\text {bias}} = {37}\text { V}$ and $U_{\text {threshold}} = {70}{\text { mV}}$ with a value of 113 ± 1 ps FWHM. These parameters are therefore applied for all following measurements. To get an impression of how the transit times in the crystal fibre depend on the absorption position, i.e. the DOI, the CTR was determined as a function of the irradiation position in a side-on DOI scan and is shown in Fig. 4.

### Double-sided readout of the 20-mm fibre

**Fig. 5:**
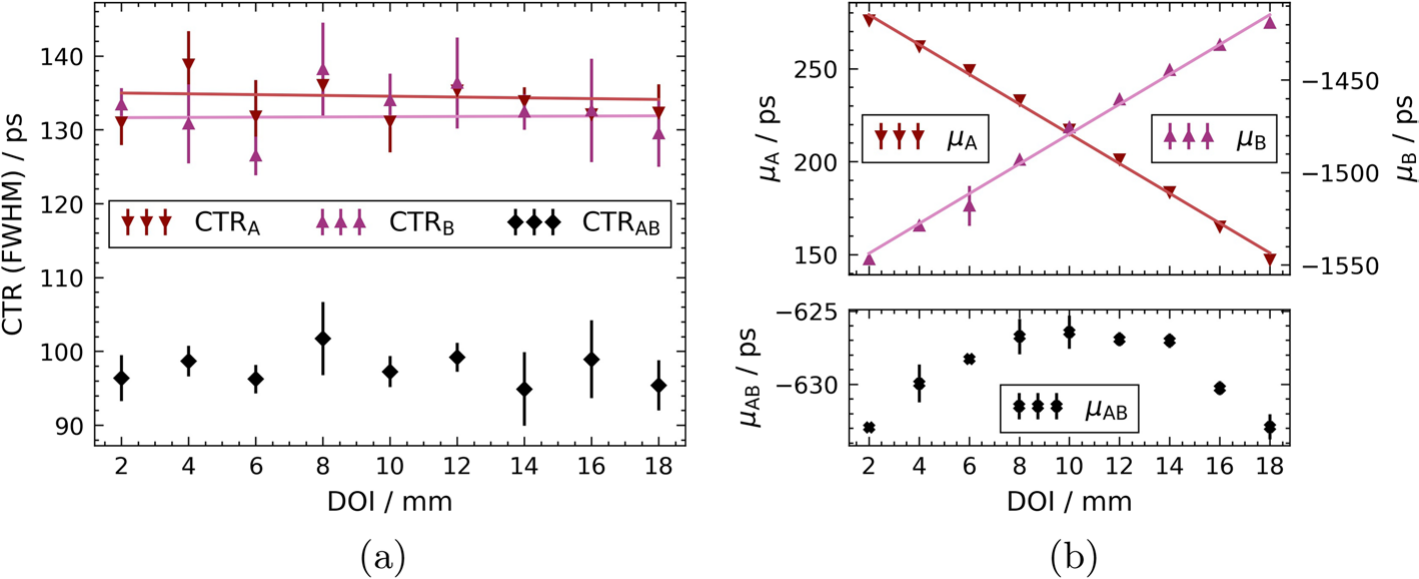
CTR and Gaussian centroids $\mu$ of the 20-mm fibre against the reference for all measured DOI positions. Linear fits are added to guide the eye.** a** CTR of the 20-mm fibre for all measured DOI positions,** b** Gaussian centroids* μ* of the 20-mm fibre against the reference for all measured DOI positions

The DOI scans performed with double-sided readout of the 20-mm fibre result in the CTR$_\text {A}$ or CTR$_\text {B}$ between the reference crystal and the SiPM A or B coupled to the fibre, as well as the CTR$_{\text {AB}}$ between the reference and the whole fibre (arithmetic mean of A and B) and the energy-weighted CTR$_{\text {AB,EW}}$. The corresponding results are shown in Fig. 5a. The average CTR for the single side readout on channel A is $\left\langle \text {CTR}_\text {A}\right\rangle = {132 \pm 1} {\text { ps}}$ and on channel B $\left\langle \text {CTR}_\text {B}\right\rangle = {135 \pm 1} {\text { ps}}$, respectively. For the use of both SiPMs and energy weighting of the timestamps, $\left\langle \text {CTR}_\text {AB,EW}\right\rangle ={101 \pm 1} {\text { ps}}$ can be achieved, or $\left\langle \text {CTR}_\text {AB}\right\rangle = {98 \pm 1} {\text { ps}}$ with equal weights. The corresponding centroids $\mu$ of the time delay distributions can be found in Fig. 5b.

**Fig. 6:**
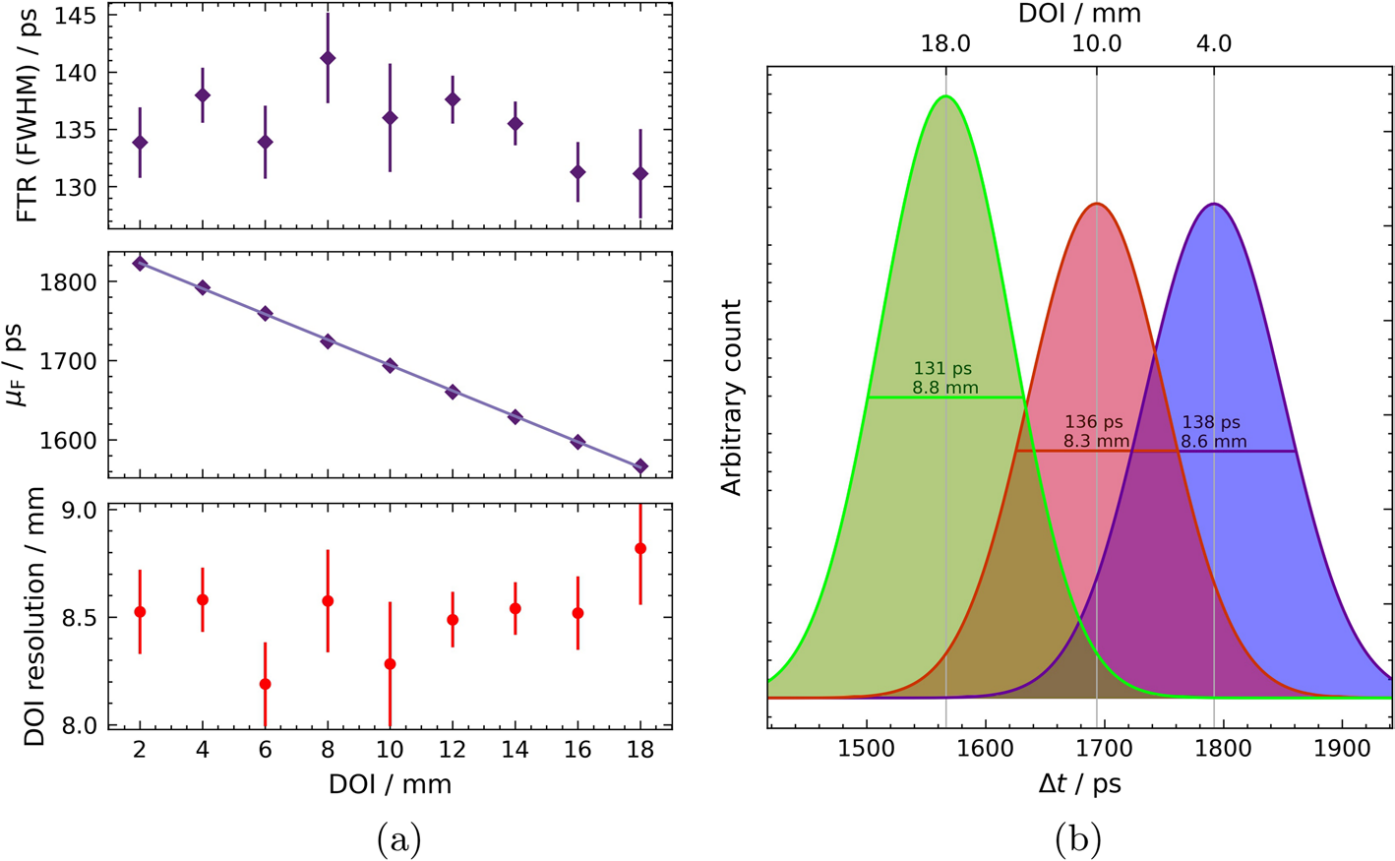
Results of the fibre-internal measurements of the 20-mm crystal. The distribution of FTR and Gaussian centroid $\mu _\text {F}$ provide information about the local DOI resolution of each irradiated DOI position. **a** FTR, Gaussian centroids *μ*_F_ of TOF histogram fits and local DOI resolution with respect to the irradiated DOI position. Linear fit is added to guide the eye, **b** DOI-specific FTRs plotted with their Gaussian fits at their centroids *μ*_F_ within the 20-mm fibre allow conclusions to be drawn about the DOI resolution

The FTR as well as the DOI-dependent progression of the Gaussian centroids, can be seen in Fig. 6a. The average FTR is $\left\langle \text {FTR}\right\rangle = {135 \pm 3} {\text { ps}}$. The slope of the Gaussian centroids $\mu _\text {F}$ is constant along the whole fibre. This linear dependency results in a constant gradient across the DOI of $\frac{\textrm{d}{\mu }}{{\text {DOI}}} = {-16.13\pm 0.24}\text { ps mm}^{-1}$. Equation (5) gives the DOI resolution within the fibre, also recorded in Fig. 6a. This local DOI resolution is almost constant over the entire course of the fibre with a spread of less than 0.7 mm and lays on average at $\left\langle \text {DOI}^\text {T}_\text {res}\right\rangle = {8.5\pm 0.2}\text { mm}$. The energy DOI resolution can also be estimated and results in $\left\langle \text {DOI}^\text {E}_\text {res}\right\rangle = {13.7\pm 0.5}\text { mm}$.

### Double-sided readout of the 100-mm scintillator

**Fig. 7:**
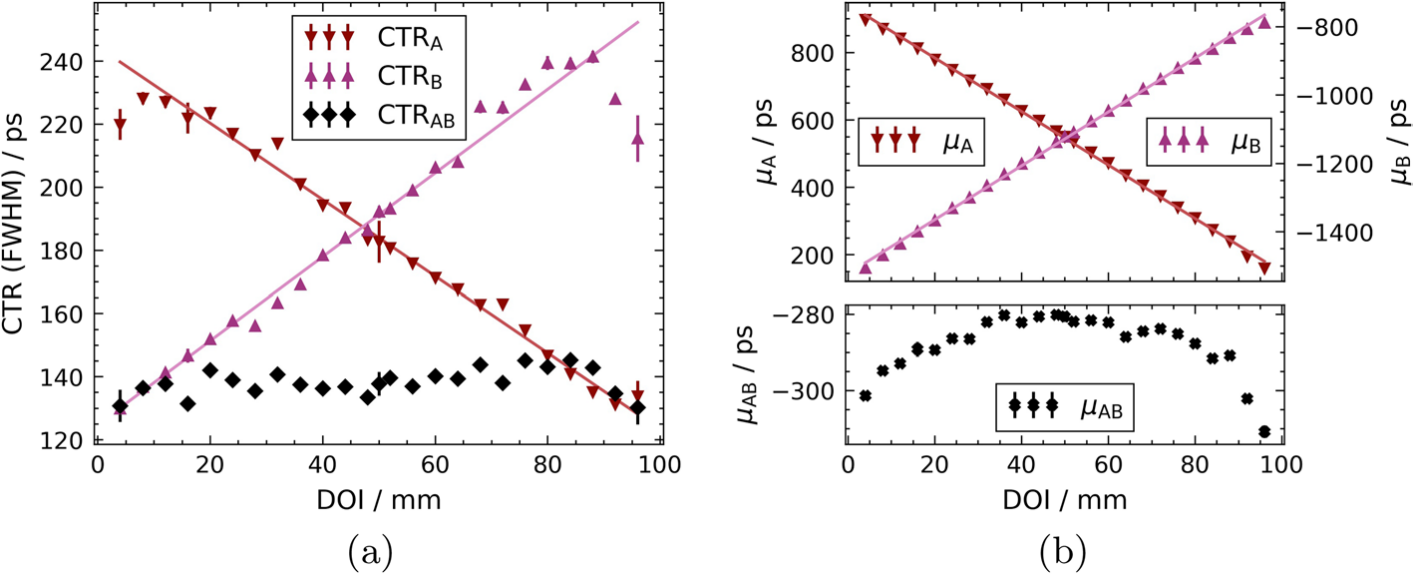
CTR and Gaussian centroids $\mu$ of the 100-mm fibre against the reference for all measured DOI positions. Linear fits are added to guide the eye.** a** CTR of the 100-mm fibre for all measured DOI positions. The averaged CTR_AB_ is independent of the DOI,** b** Gaussian centroids* μ* of the 100-mm fibre against the reference for all measured DOI positions

The CTRs and corresponding Gaussian centroids for the 100-mm fibre are shown as a function of the DOI position in Fig. 7a, b.

The average CTR for the single-side readout on channel A is $\left\langle \text {CTR}_\text {A}\right\rangle = {191 \pm 37} {\text { ps}}$ and on channel B $\left\langle \text {CTR}_\text {B}\right\rangle = {184 \pm 34} {\text { ps}}$, respectively. For the use of both SiPMs and energy weighting of the time stamps, $\left\langle \text {CTR}_\text {AB,EW}\right\rangle = {144 \pm 2} {\text { ps}}$ can be achieved, or $\left\langle \text {CTR}_\text {AB}\right\rangle = {137 \pm 1} {\text { ps}}$ with equal weights.

**Fig. 8:**
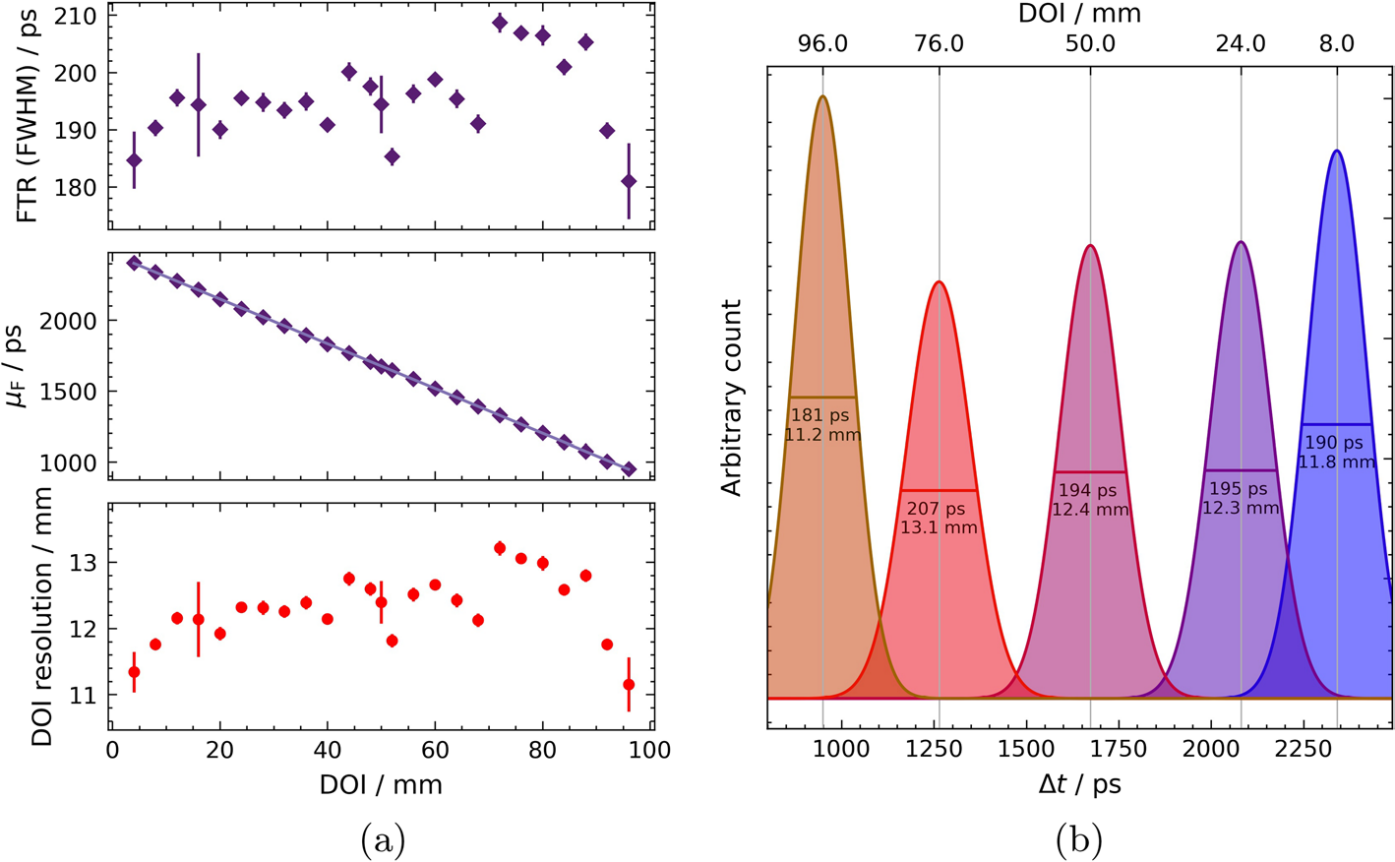
Results of the fibre-internal measurements of the 100-mm fibre. The distribution of FTR and Gaussian centroid $\mu _\text {F}$ provide information about the local DOI resolution of each irradiated DOI position.** a** FTR, Gaussian centroids *μ*_F_ of the TOF histogram fit and local DOI resolution with respect to the irradiated DOI. Linear fit is added to guide the eye.** b** DOI-specific FTRs plotted with their Gaussian fits at their centroids* μ*_F_ within the 100-mm fibre allow conclusions to be drawn about the DOI resolution

The obtained FTRs are shown in Fig. 8a. The arithmetic mean of the FTR is $\left\langle \text {FTR}\right\rangle = {195 \pm 7} {\text { ps}}$. The DOI dependence of the Gaussian centroids and the resulting local DOI resolution can be found in Fig. 8a. The distances of the Gaussian centroids $\mu$ are constant within the fibre $\mu _\text {F}$. Thus, there is a constant gradient across the DOI of $\frac{\textrm{d}{\mu }}{\text {dDOI}} = {-15.8\pm 0.02}\text { ps mm}^{-1}$. The DOI resolution can now be determined using Eq. (5). The resulting local DOI resolution for each measured DOI is plotted in Fig. 8a. On average, this results in a DOI resolution of $\left\langle \text {DOI}^\text {T}_\text {res}\right\rangle = {12.3\pm 0.5} {\text { mm}}$. An illustration can be found in Fig. 8b for selected DOIs, where the x-axis shows the specific run time differences of the individual DOI positions. In the case of the 100-mm fibre, the energy DOI resolution can be estimated to be $\left\langle \text {DOI}_\text {res}^\text {E}\right\rangle = {15.5\pm 0.6} {\text { mm}}$.

## Discussion

The best CTR of $113 \pm 1 \text { ps}$ achieved with conventional head-on single-sided readout of the 20-mm fibre at $U_\text {bias} = {37}\text { V}$ and $U_\text {thld} = {70}\text { mV}$ gives a first indication of the potential of this detector material. In this configuration, an improvement in the CTR can be seen the closer the DOI is to the open end, i.e. away from the SiPM. This effect is mainly due to the temporal close reflection of the optical photons at the open end. With the exception of the value measured at the 20-mm DOI position, a plateau occurs near the SiPM, or the CTR values drop again compared to DOIs in the middle of the fibre, which is consistent with the light propagation theory described in [13, 33]. A gamma impact in the scintillator essentially releases two photon waves that propagate in opposite directions along the scintillation fibre. At the open fibre end, the propagation wave is reflected due to the Teflon wrapping. For DOIs far away from the SiPM, the runtime difference of the two photon waves propagating (after reflection) towards the SiPM is almost zero and therefore has no influence. For DOIs close to the SiPM, the runtime difference is so large that the second wave can be filtered out with the used HF electronics and has no influence on the CTR, which, however, reduces the fast detected light to about half and therefore deteriorates timing. For DOIs in between, there is an overlap of both effects, explaining the slope.

For the double-sided readout, a different behaviour of the individual SiPMs can be observed for the 20-mm fibre with regard to the CTR. Figure 5a shows that the CTR is almost constant across all DOIs, which is due to the changed reflection conditions caused by the second SiPM on the distal fibre end. At the distal fibre end, there is no longer any reflection, so that only one photon wave at the SiPM determines the CTR, i.e. analogous to the DOIs near the SiPM in the single-sided readout configuration. The longer 100-mm fibre (see Fig. 7a), on the other hand, again shows a characteristic slope of the CTR as a function of the DOI. The CTR improves as the distance to the corresponding SiPM decreases, reaching values of around 130 ps comparable to the 20-mm fibre. At a length of 100 mm, the transparency and bulk absorption of LYSO:Ce,Ca as scintillation material has a significant influence. Light absorption and surface scattering can no longer be neglected and lead to a linear degradation of the CTR with the DOI until values of about 240 ps are reached. It is noticeable that in a range of the last 20 mm a saturation occurs at the end of the fibre far from the detecting SiPM and the CTR does not deteriorate further with increasing distance, but even improves. In this range, it can be assumed that a small fraction of the photon wave travelling away from the detecting SiPM is reflected at the far end, despite the presence of the respective SiPM, and propagates to the other end of the fibre, affecting the CTR.

It is also clear from Fig. 7a that the setup is symmetrical in nature, with both SiPMs performing equally well. The course of the CTR against the DOI shows a reciprocal slope in each case, which corresponds in its absolute value.

The resulting CTRs, when using the information from both SiPMs (compare Eq. (4)), show an almost constant value over the entire fibre for both fibre lengths, as illustrated in Figs. 5a and 7a. For the 20-mm fibre, the CTR can be significantly improved by almost a factor of $\sqrt{2}$, which is the maximum improvement considering classical photo-statistics. Compared to the head-on irradiation in a standard PET single-sided readout configuration, an improvement of more than 13 ps can be achieved, which corresponds to about 10% to 13 %. In this configuration also sub-100 ps CTRs can be measured.

The best CTR achieved by the 100-mm fibre is $\left\langle {\text {CTR}_{\rm AB}}\right\rangle = {137\pm 1} {\text { ps}}$, which corresponds to a spatial confinement of the positron-emission point along the LOR smaller than 2 cm in the system.

The closest comparable CTR value found in the literature for equal scintillator dimensions comes from Casella et al. [19] from a study with double-sided readout of $3 \times 3 \times 100 \text { mm}^{3}$ LYSO scintillators using digital SiPMs in the context of AX-PET. There, a CTR of 211 ps was achieved [19], which is 1.5 higher than the value achieved here.

Analogous to the CTR is the FTR, the coincidence time resolution between the two SiPMs of the same fibre. In both the 20-mm and 100-mm cases, as expected, there is an almost constant curve over the whole fibre, as can be seen in Figs. 6 and 8a. This is to be expected, since the total distance and thus the length that the optical signal has to travel in total to reach both SiPMs are constant. Additionally, due to the linear course of the Gaussian centroids difference $\mu _\text {F}$ (compare the middle plots in Figs. 6 and 8a), the DOI resolution results from Eq. (5) as a constant over all DOIs (see lower plots in Figs. 6 and 8a). With a time-based DOI resolution of $\left\langle \text {DOI}^\text {T}_\text {res}\right\rangle = {8.5\pm 0.2}\text { mm}$ in the 20-mm fibre or $\left\langle \text {DOI}^\text {T}_\text {res}\right\rangle = {12.3\pm 0.5}\text { mm}$ in the 100-mm fibre, these are significantly better than the corresponding energy DOI resolutions of $\left\langle \text {DOI}^\text {E}_\text {res}\right\rangle = {13.7\pm 0.5}\text { mm}$ or $\left\langle \text {DOI}_\text {res}^\text {E}\right\rangle = {15.5\pm 0.6}\text { mm}$.

**Table 1:** Summary of the main results obtained with all tested fibre configurations

Fibre	$\left\langle \text {CTR (FWHM)}\right\rangle /\text {ps}$	$\left\langle \text {FTR (FWHM)}\right\rangle /\text {ps}$	$\left\langle \text {DOI}_\text {res}^\text {T}\right\rangle /\text {mm}$	$\left\langle \text {DOI}_\text {res}^\text {E}\right\rangle /\text {mm}$
20-mm SiSi	(head-on) $113 \pm 1$	(side-on) $118 \pm 17$	n.a.	n.a.
20-mm DoSi	$98 \pm 1$	$135 \pm 3$	$8.5\pm 0.2$	$13.7 \pm 0.5$
100-mm DoSi	$137\pm 1$	$195 \pm 7$	$12.3 \pm 0.5$	$15.5 \pm 0.6$

It should be noted that the double-sided readout leads to an improved TOF capability by a much improved CTR. Furthermore, time-based double-sided readout allows good DOI separation with the resolution being slightly deteriorated if the fibre length is increased. By using the recently technologically improved NUV-MT SiPMs by Fondazione Bruno Kessler and Broadcom Inc. presented in [18], a further improvement of the CTR and thus DOI resolution is conceivable. The main results summarized in Table 1 prove the readout strategy using HF electronics and analogue SiPMs and in particular the concept of time-based double-sided readout of long fibres suitable for future TOF-PET systems.

## Conclusion

It was shown that double-sided readout of long LYSO:Ce co-doped with Ca scintillation fibres of $3 \times 3 \times 100 \text { mm}^{3}$ size can achieve state-of-the-art CTRs of 137 ps, when coupled to commercially available $3 \times 3 \text { mm}^{2}$ SiPMs from Broadcom (AFBR-S4N33C013) and using HF readout. Reducing the fibre length to 20 mm improves the time resolution to even 98 ps, a next milestone in TOF-PET. By using a solely time-based DOI estimation, the resolution is only dependent on the time resolution and shows no edge effects, which enables a uniform DOI resolution of 12.3 mm for the 100-mm-long fibre. In the 20-mm fibre, the DOI resolution measured is almost equal to 8.5 mm FWHM. The double-sided readout approach is especially interesting for applications in combined PET-MRI, where the axial arrangement allows to place the electronics “at the side” which enables an almost interference-free passage of magnetic and RF fields to the patient. A 100-mm-long fibre will allow for an electronic channel reduction of a factor 2.5, when compared to single-sided readout of 20-mm crystal, which is a standard size in PET. Increasing the fibre length would allow for even higher channel reductions, with only marginal deterioration in time and DOI resolution, due to the light-guide character of the scintillation fibre and the solely time-based estimators. Especially in brain PET, this concept could be a cost-effective approach. In this case, the axial resolution of 12 mm is easily offset by the given radial resolution of 3 mm and the high amount of parallax events expected. However, in order to truly understand the benefits and pitfalls of such an axial scanner, broader image reconstruction studies need to be conducted. Expected advancements of scintillating materials and SiPMs will allow for further improvements in time and axial (DOI) resolution. One promising field of study will be the double-sided readout of long bismuth-germanate (BGO) fibres, which could highly benefit from the prompt Cherenkov emission in terms of timing and DOI resolution and will be subject for future studies.

## Data Availability

The datasets used and/or analyzed during the current study are available from the corresponding author on reasonable request.

## Abbreviations

PET: Positron emission tomography
SNR: Signal-to-noise ratio
TOF: Time-of-flight
DOI: Depth of interaction
MRI: Magnetic resonance imaging
LYSO:Ce,Ca: Lutetium yttrium oxyorthosilicate double-doped with cerium and calcium
CTR: Coincidence time resolution
HF: High-frequency
FWHM: Full width at half maximum
LOR: Line of response
SiPM: Silicon photomultiplier
SiSi: Single-sided readout
DoSi: Double-sided readout
FOV: Field-of-view
HFR: High-frequency readout board
SPAD: Single-photon avalanche diode
HFW: Combiner board/HFwitch
BGO: Bismuth-germanate
FTR: Fibre time resolution

## References

[1] Cherry SR, Dahlbom M (2006). PET: physics, instrumentation, and scanners.

[2] Nadig V, Herrmann K, Mottaghy FM, Schulz V (2022). Hybrid total-body pet scanners-current status and future perspectives. Eur J Nucl Med Mol Imaging.

[3] Vandenberghe S, Marsden PK (2015). PET-MRI: a review of challenges and solutions in the development of integrated multimodality imaging. Phys Med Biol.

[4] Cherry SR, Jones T, Karp JS, Qi J, Moses WW, Badawi RD (2018). Total-body PET: maximizing sensitivity to create new opportunities for clinical research and patient care. J Nucl Med.

[5] Vandenberghe S, Moskal P, Karp JS (2020). State of the art in total body PET. EJNMMI Phys.

[6] Omidvari N, Jones T, Price P, Sen F, Shacklett B, Cohen S, et al. Total-body imaging of CD8+ T cells in patients recovering from COVID-19—a pilot study using the uEXPLORER total-body PET, vol 63, p. 2327. Society of Nuclear Medicine. _eprint: https://jnm.snmjournals.org/content.

[7] Vandenberghe S, Mikhaylova E, D’Hoe E, Mollet P, Karp JS (2016). Recent developments in time-of-flight PET. EJNMMI Phys.

[8] Surti S (2015). Update on time-of-flight PET imaging. J Nucl Med.

[9] Tomitani T (1981). Image reconstruction and noise evaluation in photon time-of-flight assisted positron emission tomography. IEEE Trans Nucl Sci.

[10] Prince JL, Links JM (2006). Medical imaging signals and systems.

[11] Humm JL, Rosenfeld A, Del Guerra A (2003). From PET detectors to PET scanners. Eur J Nucl Med Mol Imaging.

[12] Surti S, Karp JS (2020). Update on latest advances in time-of-flight PET. Phys Med.

[13] Gundacker S, Knapitsch A, Auffray E, Jarron P, Meyer T, Lecoq P (2014). Time resolution deterioration with increasing crystal length in a TOF-PET system. Nucl Instrum Methods Phys Res Sect A Accel Spectrom Detect Assoc Equip.

[14] angelaxiotis.: Free Base Mesh—3D model by angelaxiotis. https://skfb.ly/YZ9Z.

[15] Bisogni MG, Morrocchi M (2016). Development of analog solid-state photo-detectors for positron emission tomography. Nucl Instrum Methods Phys Res Sect A Accel Spectrom Detect Assoc Equip.

[16] Gundacker S, Turtos RM, Kratochwil N, Pots RH, Paganoni M, Lecoq P (2020). Experimental time resolution limits of modern SiPMs and TOF-PET detectors exploring different scintillators and Cherenkov emission. Phys Med.

[17] Gundacker S, Turtos RM, Auffray E, Paganoni M, Lecoq P (2019). High-frequency SiPM readout advances measured coincidence time resolution limits in TOF-PET. Phys Biol.

[18] Nadig V, Herweg K, Chou MMC, Lin JWC, Chin E, Li CA (2023). Timing advances of commercial divalent-ion co-doped LYSO: Ce and SiPMs in sub-100 ps time-of-flight positron emission tomography. Phys Med Biol.

[19] Casella C, Heller M, Joram C, Schneider T (2014). A high resolution TOF-PET concept with axial geometry and digital SiPM readout. Nucl Instrum Methods Phys Res Sect A Accel Spectrom Detect Assoc Equip.

[20] Niedźwiecki S, Białas P, Curceanu C, Czerwiński E, Dulski K, Gajos A, et al. J-PET: a new technology for the whole-body PET imaging;48(10):1567. 10.5506/APhysPolB.48.1567. 1710.11369 [hep-ex, physics:physics].

[21] Braem A, Joram C, Séguinot J, Dissertori G, Djambazov L, Lustermann W (2009). AX-PET: a novel PET detector concept with full 3D reconstruction. Nucl Instrum Methods Phys Res Sect A Accel Spectrom Detect Assoc Equip.

[22] Beltrame P, Bolle E, Braem A, Casella C, Chesi E, Clinthorne N (2011). The AX-PET demonstrator-design, construction and characterization. Nucl Instrum Methods Phys Res Sect A Accel Spectrom Detect Assoc Equip.

[23] Acerbi F, Gundacker S (2019). Understanding and simulating SiPMs. Nucl Instrum Methods Phys Res Sect A Accel Spectrom Detect Assoc Equip.

[24] Cates JW, Gundacker S, Auffray E, Lecoq P, Levin CS (2018). Improved single photon time resolution for analog SiPMs with front end readout that reduces influence of electronic noise. Phys Med Biol.

[25] Krake M, Nadig V, Schulz V, Gundacker S (2022). Power-efficient high-frequency readout concepts of SiPMs for TOF-PET and HEP. Nucl Instrum Methods Phys Res Sect A Accel Spectrom Detect Assoc Equip.

[26] Taiwan Applied Crystal. LYSO scintillation crystal. https://www.tacrystal.com/YLSO/LYSO.html.

[27] Nadig V, Yusopova M, Radermacher H, Schug D, Weissler B, Schulz V (2022). A comprehensive study on the timing limits of the TOFPET2 ASIC and on approaches for improvements. IEEE Trans Radiat Plasma Med Sci.

[28] Broadcom.: AFBR-S4N33C013: NUV-HD single silicon photo multiplier.

[29] Cargille.: Meltmount$$^{{\rm TM}}$$ instructions. https://cargille.com/wp-content/uploads/2019/03/MeltMount.pdf.

[30] Cargille.: Mounting Media—Cargille Labs. https://www.cargille.com/mounting-media/.

[31] ESPEC.: Constant Climate Cabinet LU-114. https://www.espec.co.jp/english/products/catalog/ls.pdf.

[32] Gundacker S, Auffray E, Pauwels K, Lecoq P (2016). Measurement of intrinsic rise times for various L(Y)SO and LuAG scintillators with a general study of prompt photons to achieve 10 ps in TOF-PET. Phys Med Biol.

[33] Loignon-Houle F, Gundacker S, Toussaint M, Lemyre FC, Auffray E, Fontaine R (2021). DOI estimation through signal arrival time distribution: a theoretical description including proof of concept measurements. Phys Med.

